# The signal quality of tripolar Laplacian electrogram compared to bipolar electrogram in cardiac electrophysiology

**DOI:** 10.1002/joa3.70101

**Published:** 2025-05-29

**Authors:** Soichiro Yamashita, Makoto Takemoto, Wataru Fujimoto, Koji Kuroda, Junichi Imanishi, Masamichi Iwasaki, Takafumi Todoroki, Masanori Okuda

**Affiliations:** ^1^ Department of Cardiology Hyogo Prefectural Awaji Medical Center Sumoto Japan

**Keywords:** atrial tachycardia, electroanatomical mapping, electrophysiology, far‐field potential, tripolar Laplacian electrogram

## Abstract

**Background:**

The usefulness of tripolar Laplacian electrogram (TLE) in cardiac electrophysiology is unknown.

**Objective:**

We investigated the difference in the morphology, voltage amplitude, noise level, and far‐field potential (FFP) between TLE and bipolar electrogram (BE).

**Methods:**

Twenty‐two patients who underwent catheter ablation for atrial fibrillation were analyzed. TLE and BE were simultaneously obtained using 64‐electrode basket catheters during atrial tachycardia or pacing. Local electrograms (EGMs) were analyzed at the locations of three activation patterns: normal conduction, wavefront collision, and slow conduction (SC). In addition, each activation pattern was analyzed at two different wavefront angles: horizontal and vertical, respectively. The voltage amplitude, duration, morphology, and deflection number were compared. The noise level and FFP amplitude were also analyzed.

**Results:**

With all activation patterns and wavefront angles, the voltage amplitude was significantly lower on TLE than on BE, and the mean amplitude ratio of TLE/BE was 0.75. The duration and deflection number of EGM were comparable. The ventricular FFP amplitude was lower on TLE than on BE (0.04 mV vs. 0.40 mV, *p* = .01). TLE also had less noise than BE (0.08 mV vs. 0.15 mV, *p* = .02), with a noise reduction of 47%. At the SC area, low‐voltage EGMs below the mean noise levels were more common with BE than with TLE (29% vs. 8%, *p* = .04).

**Conclusion:**

TLE has a lower voltage amplitude than BE for all activation patterns and wavefront angles, and it removes more noise and FFPs. TLE is useful for analyzing low‐voltage and fractionated EGMs regardless of the wavefront angle.

## Graphical abstract

Tripolar Laplacian electrogram (TLE) is one of the multipolar electrograms. TLE has a lower voltage amplitude than bipolar electrograms for all activation patterns and wavefront angles, and it removes more noise and far‐field potentials. TLE is useful for analyzing low‐voltage and fractionated electrograms regardless of the wavefront angle.

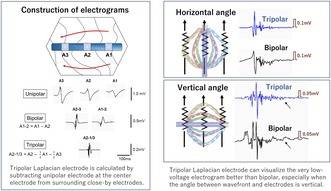

## 1 INTRODUCTION

**FIGURE 1:**
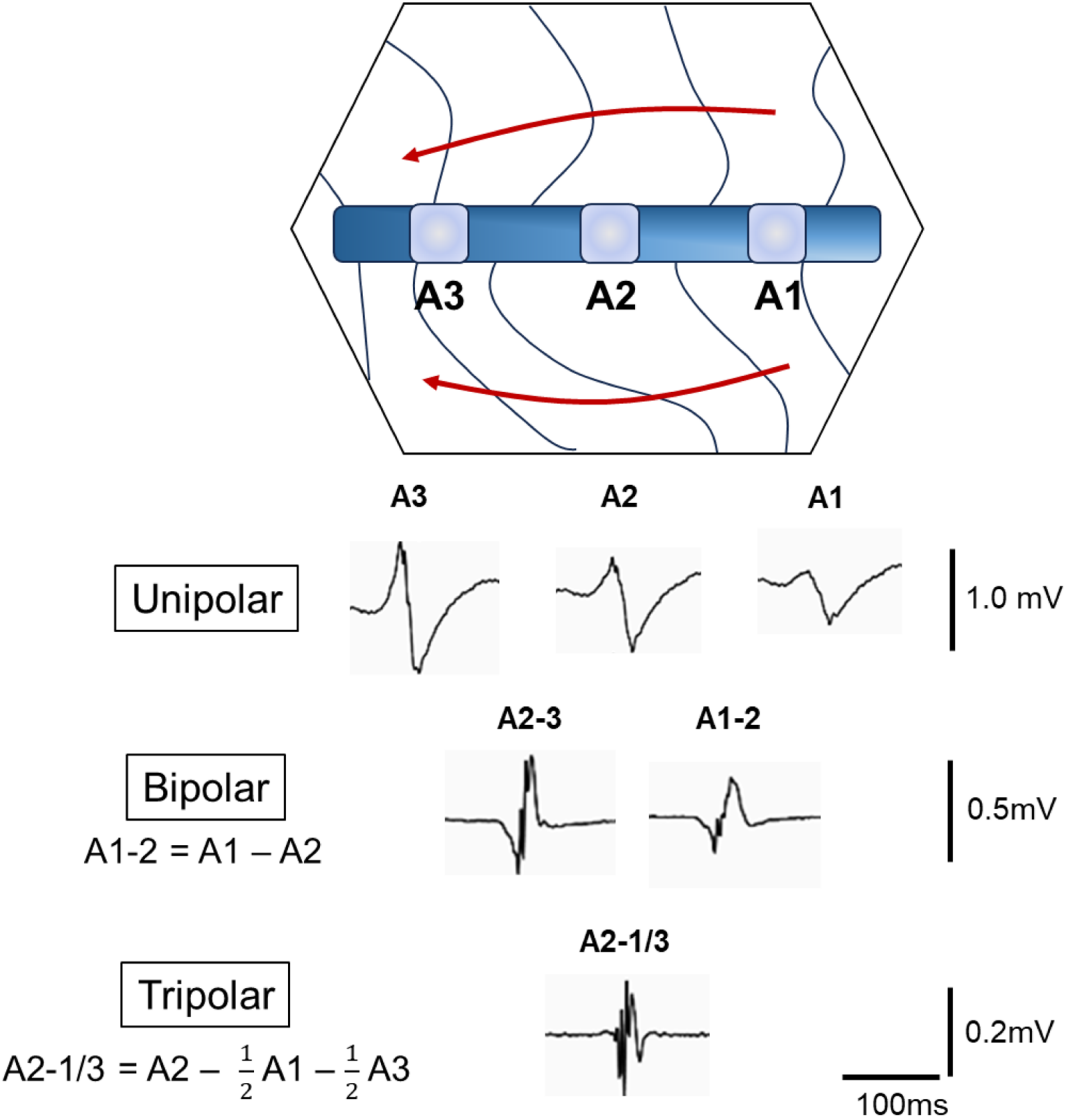
The concept of EGM construction. This figure shows the electrode catheter being used to create an activation map. Unipolar EGMs are recorded at each of the three electrodes (electrodes A1 to A3). Bipolar EGMs consist of a subtraction between two unipolar electrodes. The corresponding tripolar EGM is calculated by subtracting the unipolar EGM at the center electrode from the surrounding close‐by electrodes. EGM, electrogram.

Electrogram (EGM) is an essential tool to identify and treat arrhythmias. Bipolar EGM (BE) is widely used for electroanatomical mapping because of its lower sensitivity to far‐field potentials (FFPs) and repolarization waves than unipolar EGM. However, BE has some disadvantages. Specifically, the amplitude depends on the wavefront direction, and the morphology depends on the interelectrode spacing, conduction velocity, and wavefront direction, which is called “bipolar blindness.”^1^ Tripolar Laplacian electrogram (TLE) overcomes these limitations of BE. TLE is calculated by subtracting unipolar EGM at the center electrode from surrounding close‐by electrodes.^2^, ^3^ TLE is reported to be superior to BE in that it reduces the noise level, is insensitive to the wavefront orientation, and has high spatial resolution.^3^, ^4^, ^5^, ^6^, ^7^ Moreover, simulation studies have reported that the far‐field rejection of the tripolar configuration is greater than that of the bipolar configuration.^8^ TLE has been used in some medical instruments, such as body surface mapping and electroencephalography.^9^, ^10^ In cardiac electroanatomical mapping, TLE can be used in the RHYTHMIA™ system. When mapping is performed by the system, all potentials are simultaneously recorded in unipolar, bipolar, and TLE. TLE is constructed by subtracting the unipolar EGM of two adjacent electrodes from the unipolar EGM of one electrode. The construction of EGMs including unipolar, bipolar, and TLE is shown in Figure 1. TLE is used to analyze low‐amplitude potentials and complicated activation patterns, which is called a LUMIPOINT module. The usefulness of the LUMIPOINT has been reported in various situations including the detection of a slow conduction area,^11^ a circuit isthmus of reentrant tachycardia,^12^ and a trigger of atrial fibrillation.^13^ However, it is not clear how the morphologies of EGMs differ between TLE and BE. The aim of this study was to compare TLE with BE with respect to EGM morphology and voltage amplitude by wavefront direction.

## 2 METHODS

### 2.1 Study population

Consecutive patients who underwent catheter ablation for atrial fibrillation from March 2024 to November 2024 at Hyogo Prefectural Awaji Medical Center were included in this study. Of these, patients who had an electroanatomical map created using RHYTHMIA (Boston Scientific, MA, United States) during atrial tachycardia (AT) or atrial pacing were included. Their maps were retrospectively analyzed. All procedures were performed under general anesthesia with a supra‐glottic airway device. Antiarrhythmic drugs were stopped more than five half‐lives before the procedure. The study was approved by the local ethics committee of our institution, and all patients provided written informed consent. The study was performed in accordance with the principles outlined in the Declaration of Helsinki.

### 2.2 Mapping system

In all cases, RHYTHMIA was used for data collection. The Orion catheter (64‐electrode basket catheter, Boston Scientific) was used to create a shell of the cardiac chamber and record EGMs. TLE and BE were filtered at 30 and 300 Hz, and unipolar EGM was at 1 and 300 Hz. In all cases, we used the inferior vena cava as a unipolar reference. Steerable sheaths were used to stabilize the catheter control and to reliably obtain the potential. Atrial pacing for mapping was derived from a catheter inserted into the coronary sinus (BeeAT, Japan Lifeline, Tokyo, Japan) at 80–100 bpm. TLEs and BEs were simultaneously obtained during AT or pacing with the following beat acceptance criteria: cycle length, delta R (difference in activation time between two electrodes), respiration phase, catheter stability, and tracking quality. Both types of EGM were analyzed offline and compared retrospectively.

### 2.3 Activation pattern analysis

**FIGURE 2:**
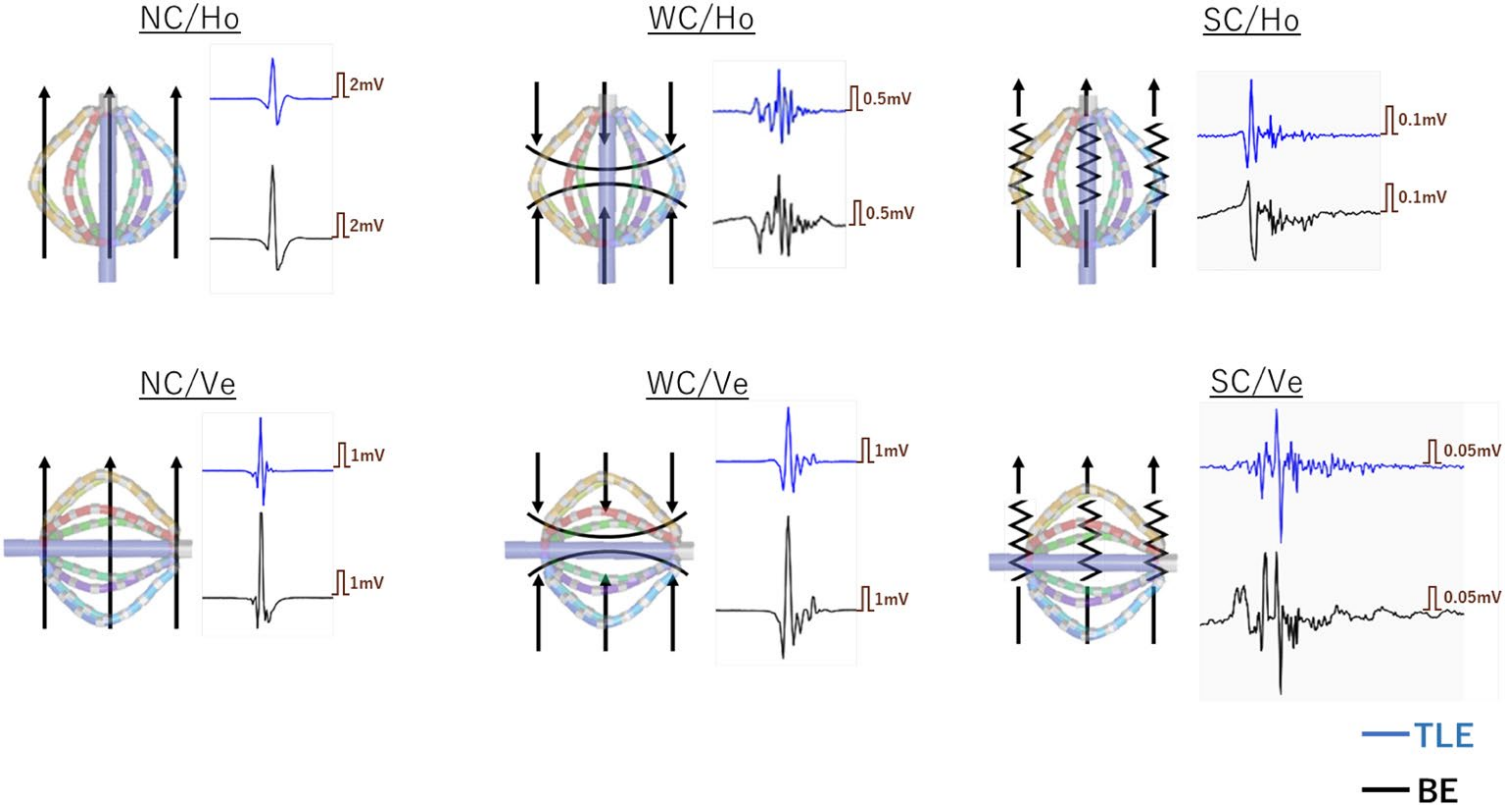
The six phenotypes were determined according to the activation pattern and the relationship between the direction of the wavefront and the catheter electrodes (wavefront angle). The black arrows indicate the wavefront direction. Representative potentials for each type are shown on their right side. The blue tracing indicates TLE and the black tracing indicates BE. BE, bipolar electrogram; Ho, horizontal; NC, normal conduction; SC, slow conduction; TLE, tripolar Laplacian electrogram; Ve, vertical; WC, wavefront collision.

In the acquired maps, locations of three activation patterns were retrospectively identified: normal conduction (NC), wavefront collision (WC), and slow conduction (SC). WC was defined when two wavefronts collided from different directions. SC was defined when the wavefront propagated linearly and the conduction velocity was less than 0.5 m/s, as previously reported.^14^ The conduction velocity was measured from the distance and time between two local points on the activation map. Areas of linear propagation with normal conduction velocity were defined as NC areas. These definitions of electrophysiological analysis were based on a previous report.^14^ In addition, the three activation patterns were classified into horizontal (Ho) and vertical (Ve) angles according to the angle between the direction of propagation and the electrodes. The angle of the wavefront to the long axis of the Orion mapping catheter at the time the potential was acquired was measured using a protractor. The angle from −15° to 15° was defined as Ho, and from 75° to 105° was defined as Ve. Other angles were excluded from the analysis. Local EGMs were analyzed in three activation patterns (NC/WC/SC) and two angles (Ho/Ve), for a total of six patterns. Representative local EGMs for each pattern are shown in Figure 2. These patterns were visually identified on the RHYTHMIA system and analyzed by two electrophysiologists. All manual analyses were evaluated in consensus by two authors (S.Y. and M.T., with 14 and 10 years of experience in cardiac electrophysiology, respectively).

### 2.4 Comparison of TLE and BE

TLE and BE were simultaneously obtained and retrospectively compared offline using the RHYTHMIA system. For each activation pattern, we selected local EGMs from which three consecutive beats could be recorded. The voltage amplitude, duration, morphology, and number of deflections were measured and averaged over the three beats. Then they were compared between TLE and BE for each activation pattern.

**FIGURE 3:**
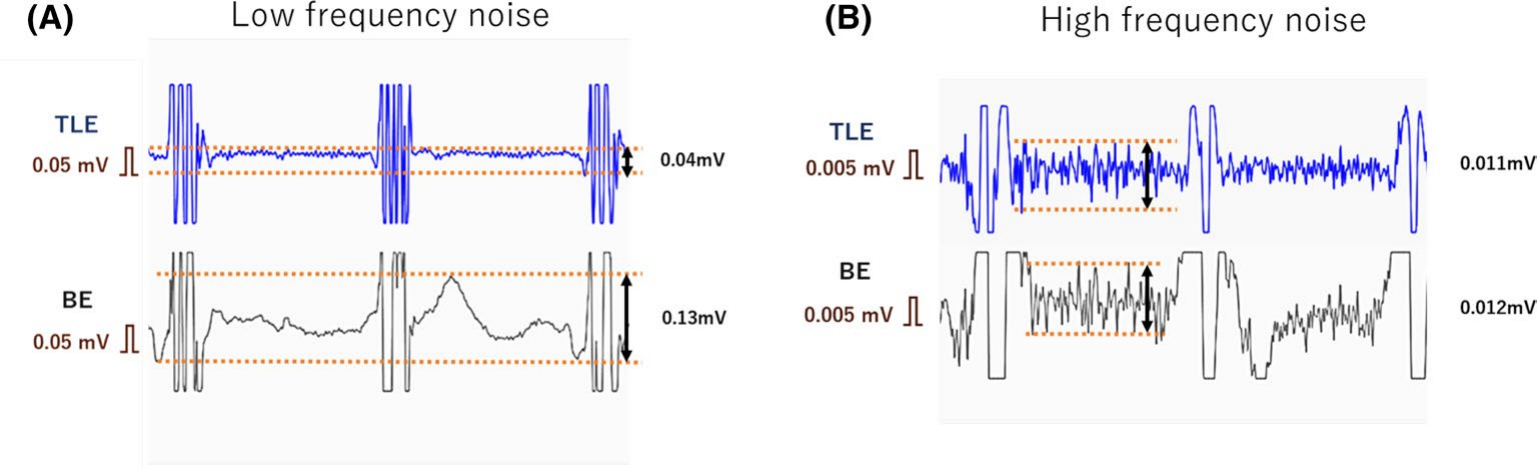
Method for measuring the noise level. The blue tracing indicates TLE and the black tracing indicates BE which are simultaneously recorded. They were observed for 1 s at the point acquired by the stationary catheter under general anesthesia. (A) The level of low frequency noise was defined as the difference between the peak and nadir of drift in the isoelectric line. (B) The level of high frequency noise was defined as the difference between the peak and nadir of a high frequent oscillation at isoelectric line. They were measured at the isoelectric line between the R‐R interval where the influence of LFN was minimal. BE, bipolar electrogram; TLE, tripolar Laplacian electrogram.

For the assessment of noise, we classified and analyzed the noise according to its frequency. The level of low‐frequency noise (LFN) was defined as the difference between the peak and nadir of drift in the isoelectric line (Figure 3A). Drift was observed for 1 s at the point acquired by the stationary catheter under general anesthesia. Simultaneously, high‐frequency noise (HFN) was measured at the isoelectric line between the R‐R interval where the influence of LFN was minimal. The level of HFN was defined as the difference between the peak and nadir of a high‐frequency oscillation at the isoelectric line (Figure 3B). Noise resulting from obvious electrode contact and movement artifact was excluded. We manually measured LFN at a sensitivity of 0.05 mV and HFN of 0.005 mV. The amplitude of LFN and HFN was respectively compared to the corresponding BE and TLE. We also analyzed the amplitude of the noise divided by the pulse amplitude. The pulse amplitude is what the system automatically measured as the voltage amplitude of the local EGM.

**FIGURE 4:**
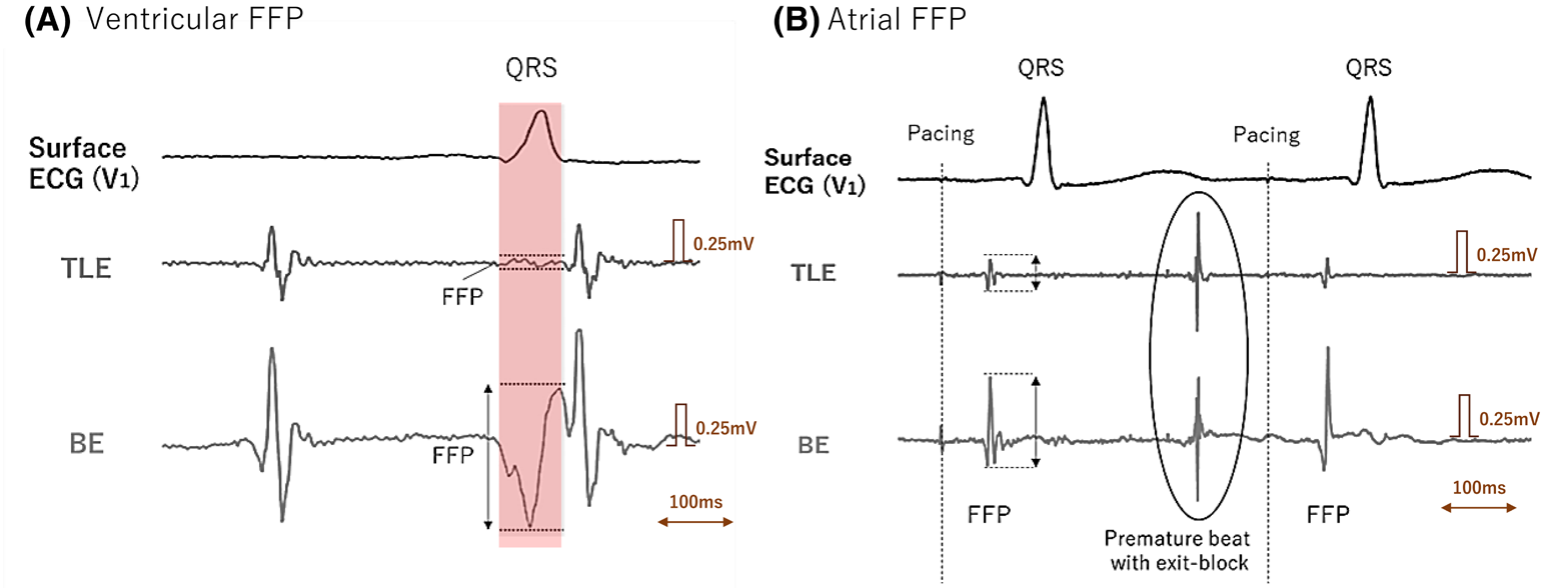
Method of measuring FFPs. (A) Ventricular FFP. The mapping catheter was located at the anterior side of the mitral annulus during atrial tachycardia. Three tracing indicates the surface ECG, TLE, and BE that were simultaneously recorded. The FFP of a ventricular potential was coincided with the timing of the QRS complex (red shadow), and it could be identified by comparing them with other beats. (B) Atrial FFP. The mapping catheter was located at electrically isolated right superior PV during atrial pacing. The FFPs of a right atrium were identified in isolated PV. The premature beat from the PV showed an exit‐block to atrium, indicating the PV isolation was completed. BE, bipolar electrogram; CS, coronary sinus; ECG, electrocardiogram; FFP, far‐field potential; PV, pulmonary vein; TLE, tripolar Laplacian electrogram.

FFPs were evaluated for atrial and ventricular types, and the differences between TLE and BE were compared. To measure the ventricular FFP, we used a far‐field ventricular potential that can be recognized near the mitral annulus (Figure 4A). Ventricular FFP was defined as a low‐frequency potential that did not coincide with the timing of atrial excitation but that did coincide with the timing of the QRS complex. Meanwhile, atrial FFP was defined as the remaining FFP in electrically isolated pulmonary veins (Figure 4B). Pulmonary vein isolation was confirmed by exit‐block from pacing in the pulmonary vein, or by dissociated beats isolated in the pulmonary vein. We compared the voltage amplitude of FFPs that were simultaneously recorded in TLE and BE.

### 2.5 Statistical analysis

Continuous data are presented as the mean ± standard deviation. Differences between mean data were compared using the Student's *t*‐test for variables with a Gaussian distribution. For variables with a non‐Gaussian distribution, the data were compared by the Mann–Whitney *U* test for independent samples, or by Wilcoxon's nonparametric test for paired samples. Categorical data are presented as percentages, and differences in proportions were identified using the chi‐square test. *R* ^2^ values for the correlations of nonlinearly distributed values were obtained using the linear least‐squares fit of log‐transformed data. For all statistical tests, *p* < .05 was considered statistically significant. The statistical analyses were performed using SPSS version 20.0 software (Chicago, IL, USA).

## 3 RESULTS

### 3.1 Study population

Total 22 patients and 32 maps were included in the analysis. Their mean age was 74 ± 3 years and 13 patients were male. The mean left atrial diameter and left atrial volume index were 47 mm and 51.1 mL/m^2^, respectively. Eight patients had previously undergone catheter ablation for AF. The mean CHA2DS2‐VASc score was 4.4 ± 1.1. The summary of patient characteristics is shown in Table 1.

**TABLE 1:** Baseline patient characteristics.

Characteristics	*n* = 22
Age	74 ± 3
Male	13 (59%)
CHA2DS2‐VASc score	4.4 ± 1.1
Left Atrial Volume Index, mL/m^2^	51.1 ± 5.5
Coronary artery disease	5 (23%)
Hypertension	13 (59%)
Diabetes mellitus	4 (18%)
Obstructive sleep apnea syndrome	4 (18%)
Valvular heart disease	7 (32%)
History of cardiac surgery	2 (9%)
History of catheter ablation	8 (36%)
Pulmonary vein isolation	8 (36%)
Peri‐mitral linear ablation	3 (14%)
Left posterior wall isolation	6 (27%)
Peri‐tricuspid linear ablation	4 (18%)
Others	4 (18%)
Medications	
Sodium channel blocker	0
Beta‐blocker	3 (14%)
Amiodarone	0
Nondihydropyridine calcium‐channel blocker	4 (18%)

The mean mapping points were 12,433 ± 1289. Voltage maps revealed that the low‐voltage area and scarring were found in 15 of 32 maps. Twenty‐four maps were created under atrial pacing, while eight were under AT. Of the patients with AT, three had peri‐mitral reentrant tachycardia, three had peri‐tricuspid reentrant tachycardia, and two had focal tachycardia in the left atrium. All ATs were mapped after performing ablation. Of those, four ATs were induced by atrial rapid pacing, and the residual four were mapped with AF changed to AT by ablation. Mapping data are summarized in Table S1.

### 3.2 Electrocardiogram characteristics for each activation pattern

Fourteen cases were excluded because of angle mismatch, and two areas were excluded because of lack of potential stability. As a result, total of 105 areas of interest were analyzed. Of these, 25 areas (24%) were identified as NC/Ho, and 25 (24%) were NC/Ve; 13 (12%) were WC/Ho, and 14 (13%) were WC/Ve; and 15 (14%) were SC/Ho, and 13 (12%) were SC/Ve. For all activation patterns and angles, the voltage amplitude was significantly lower on TLE than on BE. The mean amplitude ratio of TLE/BE was 0.75 ± 0.04. The duration of EGM was comparable among all activation patterns/wavefront angles, with the exception of SC/Ve, for which the duration was longer on TLE than on BE (79 ± 24 ms vs. 75 ± 23 ms, *p* < .01). The deflection number of EGM was also comparable between TLE and BE for all patterns except for SC/Ve. For SC/Ve, TLE had more deflections than BE (15.3 ± 3.5 vs. 12.2 ± 2.9, *p* < .01). The results are summarized in Table 2.

**TABLE 2:** Characteristics of TLE and BE for each activation pattern and wavefront angle.

Activation pattern	Wavefront angle	Voltage amplitude (ms)		*p*	Duration (ms)		*p*	Deflection (*n*)		*p*
		TLE	BE		TLE	BE		TLE	BE	
NC (*n* = 50)	Vertical (*n* = 25)	1.22 ± 0.74	1.56 ± 1.09	.02	35 ± 14	34 ± 16	NS	4.2 ± 0.9	3.8 ± 1.3	NS
	Horizontal (*n* = 25)	1.59 ± 1.11	2.01 ± 1.33	.02	31 ± 7	31 ± 8	NS	3.7 ± 1.8	4.0 ± 1.5	NS
WC (*n* = 27)	Vertical (*n* = 13)	2.61 ± 2.01	3.23 ± 2.33	.02	36 ± 9	38 ± 11	NS	6.3 ± 1.2	6.0 ± 3.5	NS
	Horizontal (*n* = 14)	0.79 ± 0.77	1.14 ± 1.03	.02	49 ± 22	48 ± 23	NS	6.5 ± 2.5	5.3 ± 2.6	NS
SC (*n* = 28)	Vertical (*n* = 15)	0.26 ± 0.11	0.33 ± 0.12	.04	79 ± 24	75 ± 23	.01	15.3 ± 3.5	12.2 ± 2.9	.01
	Horizontal (*n* = 13)	0.20 ± 0.09	0.27 ± 0.14	.04	80 ± 12	81 ± 16	NS	12.6 ± 5.3	12.0 ± 5.2	NS

Abbreviations: BE, bipolar electrogram; NC, normal conduction; SC, slow conduction; TLE, tripolar Laplacian electrogram; WC, wavefront collision.

### 3.3 Processing of noise and FFP

Of all 105 areas, 21 areas were excluded because of the artifacts by electrode contacts and movements. The residual 84 areas were analyzed. In LFN analysis, TLE showed lower noise levels than BE (0.08 ± 0.03 mV vs. 0.15 ± 0.05 mV, *p* = .02). The LFN to pulse amplitude ratio was also lower on TLE than on BE (3.2% ± 0.1% vs. 8.1% ± 0.7%, *p* = .01). Overall, TLE had a noise reduction of 47% compared with BE. On the other hand, there was no difference in HFN levels between TLE and BE (0.005 ± 0.004 mV vs. 0.008 ± 0.004 mV, *p* = .41). HFN to pulse amplitude was also comparable (0.2% ± 0.2% vs. 0.4% ± 0.2%, *p* = .18). The results are shown in Table 3.

**TABLE 3:** Noise and FFP in TLE and BE.

	TLE	BE	*p*
Areas of interest (*n* = 84)			
HFN amplitude, mV	0.005 ± 0.004	0.008 ± 0.004	.41
HFN/pulse amplitude	0.2% ± 0.2%	0.4% ± 0.2%	.18
LFN amplitude, mV	0.08 ± 0.03	0.15 ± 0.05	.02
LFN/pulse amplitude	3.2% ± 0.1%	8.1% ± 0.7%	.01
Ventricular FFP, mV (*n* = 30)	0.04 ± 0.08	0.40 ± 0.28	.01
Atrial FFP, mV (*n* = 30)	0.02 ± 0.01	0.08 ± 0.03	.02

Abbreviations: BE, bipolar electrogram; FFP, far‐field potential; HFN, high frequency noise; LFN, low frequency noise; TLE, tripolar Laplacian electrogram.

Ventricular and atrial FFPs were analyzed in 30 areas of interest each. FFPs were significantly lower in TLE than BE, both in ventricular FFP (0.04 ± 0.08 mV vs. 0.40 ± 0.28 mV, *p* = .01) and atrial FFP (0.02 ± 0.01 mV vs. 0.08 ± 0.03 mV; *p* = .02). Compared to BE, TLE reduced ventricular FFP by 91% and atrial FFP by 77%.

### 3.4 Resolution of low‐voltage electrogram

**FIGURE 5:**
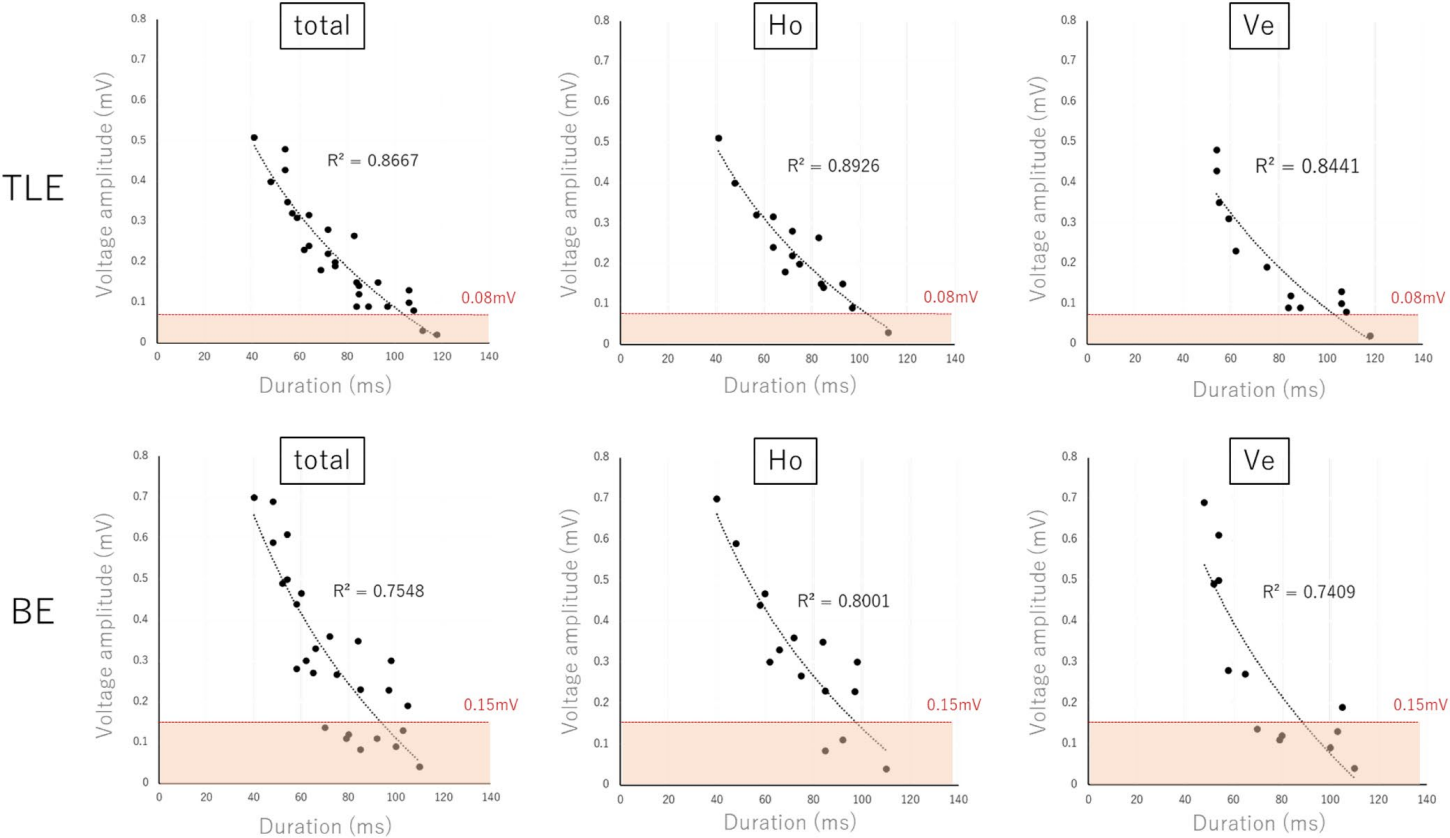
There was an inverse relationship between the voltage amplitude and the EGM duration at the SC area in various conditions. The pink shadows indicate values below the average level of drift noise (0.08 mV for TLE, 0.15 mV for BE). The pink shadow indicates points with lower noise levels that may be difficult to identify. Low‐voltage EGMs below the mean noise levels were more common for BE than for TLE (29% vs. 8%, *p* = .04). The *R* ^2^ value for the correlation between the voltage and EGM duration was lowest for BE/Ve. BE, bipolar electrogram; EGM, electrogram; Ho, horizontal; SC, slow conduction; TLE, tripolar Laplacian electrogram; Ve, vertical.

The relationships between the voltage amplitude and duration of EGM at SC areas in various conditions are shown in Figure 5. The graphs are divided by TLE/BE and Ho/Ve combinations, respectively. For all activation patterns, an inverse relationship was identified between the duration and voltage of SC areas; the higher the voltage, the shorter the duration. The *R* ^2^ value for the correlation was higher for TLE than for BE (0.87 vs. 0.76). Low‐voltage EGMs below the mean noise levels were more common for BE than for TLE (29% vs. 8%, *p* = .04).

## 4 DISCUSSION

This is the first study to report the characteristics of TLE in cardiac electrophysiology. This study showed that (1) TLE had a lower voltage amplitude than BE, regardless of the wavefront angle and activation pattern, and (2) FFP and noise were suppressed to low levels on TLE. As a result, TLE had better resolution for low‐voltage fractionated EGMs. In addition, (3) the EGM morphology was comparable for all activation patterns except for SC/Ve. For SC/Ve, TLE had a longer duration and more deflections than BE.

Novel multipolar EGMs have been developed and their usefulness has been reported.^15^, ^16^, ^17^, ^18^ The advantages of multipolar EGM are the insensitivity to remote electrical activity, and less wavefront direction dependent than bipolar. The greatest disadvantage of multipolar EGM is the loss of spatial resolution.^16^ In addition to TLE, omnipolar and multipolar mapping are offered in other commercial mapping systems and some clinical benefits have been reported; the accuracy of voltage and activation maps,^17^ and high specificity for the detection of low voltage and scar.^15^ They have different properties because of their different calculation methods, although no studies have compared them. Omnipolar mapping (Abbott, MN, USA) is calculated within a clique, which is defined as a square of four electrodes from which the bipolar EGM with the largest amplitude is extracted.^15^ Meanwhile, the basic concept of multipolar mapping (Biosense Webstar, CA, USA) is the reduction of FFP in unipolar EGM.^17^ FFP can be recognized by comparison of EGM with the surrounding electrodes. Once identified, the FFP component is subtracted from that electrode unipolar EGM. TLE in RHYTHMIA system may be superior to omnipolar and multipolar in terms of spatial resolution because it is estimated with very short electrode spacing. Further investigations are needed to understand the difference.

### 4.1 Assessment of fractionated electrogram

Electrical remodeling and fibrosis cause significant changes to EGM waveforms. SC areas feature a slow conduction velocity and low voltage amplitude and fractionation, which reflect the characteristics of fibrosis.^19^ The identification of SC areas is critical for the treatment of reentrant tachyarrhythmias. Although visualization of very small SC potentials requires a high resolution, noise can be an obstacle to this. As shown in our study, TLE was superior to BE in this respect and was better suited for SC analysis. TLE had a lower voltage amplitude than BE, but it reduced the noise level to a greater degree, resulting in better interpretation of very small EGMs. Figure S1 shows a representative case of a 78‐year‐old female with AT, which demonstrates the advantages of TLE. Low‐amplitude fractionated EGMs were identified at SC/Ve areas in the left atrial anterior wall. By raising the resolution, drift noise became more pronounced on BE, making it difficult to identify the whole EGM. Conversely, on TLE, small and fractionated EGMs were still identifiable with a reproducibility.

### 4.2 Wavefront angles and electrodes

In the SC/Ve pattern (but not in the SC/Ho pattern), TLE showed a longer EGM duration and more deflections than BE. This result indicated that such potentials can only be expressed by TLE. With BE, a wavefront that is perpendicular to the two electrodes does not register as a larger deflection because there is no time difference between when the wavefront hits both electrodes, which is called “bipolar blindness.”^20^, ^21^, ^22^ Using TLE minimizes the likelihood that a perpendicular wavefront will cancel. Additionally, the signal morphology is the same for a tripolar signal from three vertically aligned electrodes whether the wavefront propagates from the top to the bottom or the bottom to the top. BE may not represent all potentials and may underestimate them, especially at the vertical angle. In addition, the *R* ^2^ value for the correlation between the voltage and duration of EGM was lowest for the BE/Ve pattern at the SC area (Figure 5). This also indicated that BE was more vulnerable to vertical wavefronts than TLE.

### 4.3 Practical use of TLE

The LUMIPOINT module in the RHYTHMIA system identifies fractionated EGMs using TLE, and its usefulness has been reported in various situations, including detection of the circuit isthmus of AT^11^ and ventricular tachycardia,^12^ as well as detection of the fractionated signal area as an origin of atrial fibrillation,^13^, ^23^ local abnormal ventricular activity,^24^ and slow atrioventricular node pathways.^25^ TLE has also been used to analyze complex EGMs and cycle length during the mapping of atrial fibrillation.^26^ Because atrial fibrillation mapping requires analysis of complicated and fine patterns, TLE is useful because it eliminates the effects of FFPs and wavefront angles, and it can capture low‐voltage fractionated EGMs.

There are several disadvantages in TLE. As with other multipolar EGMs, TLE has lower spatial resolution than BE.^16^ TLE in RHYTHMIA is calculated with electrodes on one spline and is close to two‐dimensional. Compared to other multipolar EGMs, the resolution is higher, but may be a little more susceptible to vectors. Furthermore, TLE has some unsolved issues including the lack of information on the optimal cutoff value for the low voltage zones, and a limitation of the mapping catheter that can be used. Further investigations are needed.

### 4.4 Study imitations

This study has several limitations. First, this study analyzed only EGMs acquired with the Orion catheter. With a decrease in the interelectrode distance, the TLE amplitude increases.^3^ Therefore, if other electrode catheters are used, the results could vary depending on the electrode spacing and size. Second, all activation patterns were created using BE. Although it is a widely used clinical technique, there may be limitations to annotation with BE for complicated activations. Third, the angles between two electrodes and wavefronts were only examined horizontally and vertically. The other vectors were not analyzed. Fourth, although the reproducibility was confirmed by two electrophysiologists, the unblinded nature of this study may have influenced the data collection. Moreover, manual measurements might involve a certain inaccuracy.

Finally, the study included a small sample size.

## 5 CONCLUSIONS

TLE had a lower voltage amplitude than BE for all activation patterns and wavefront angles. However, TLE was more useful for analyzing low‐voltage and fractionated EGMs than BE because it could reduce noise and FFPs. Although the morphology of TLE was equivalent to that of BE, some potentials, such as SC/Ve, were only identifiable on TLE.

## FUNDING INFORMATION

This research did not receive any specific grant from funding agencies in the public, commercial, or not‐for‐profit sectors.

## CONFLICT OF INTEREST STATEMENT

The authors declare no conflicts of interest.

## ETHICS STATEMENT

The study was fully anonymous, and no sensitive data were collected or managed. The project was conducted in accordance with local laws and regulations.

## CONSENT

The authors have nothing to report.

## PATIENT CONSENT STATEMENT

Patient consent for publication was obtained.

## Supporting information


Figure S1.

Table S1.

Data S1.

## Data Availability

The dataset generated and analyzed in this project is not publicly available but is available from the corresponding author at a reasonable request.
