# Supplementary figures and images for: The signal quality of tripolar Laplacian electrogram compared to bipolar electrogram in cardiac electrophysiology
Source: J Arrhythm. 2025 May 29;41(3):e70101. doi: 10.1002/joa3.70101 (PMC12120260; doi:10.1002/joa3.70101)

## Slide 1
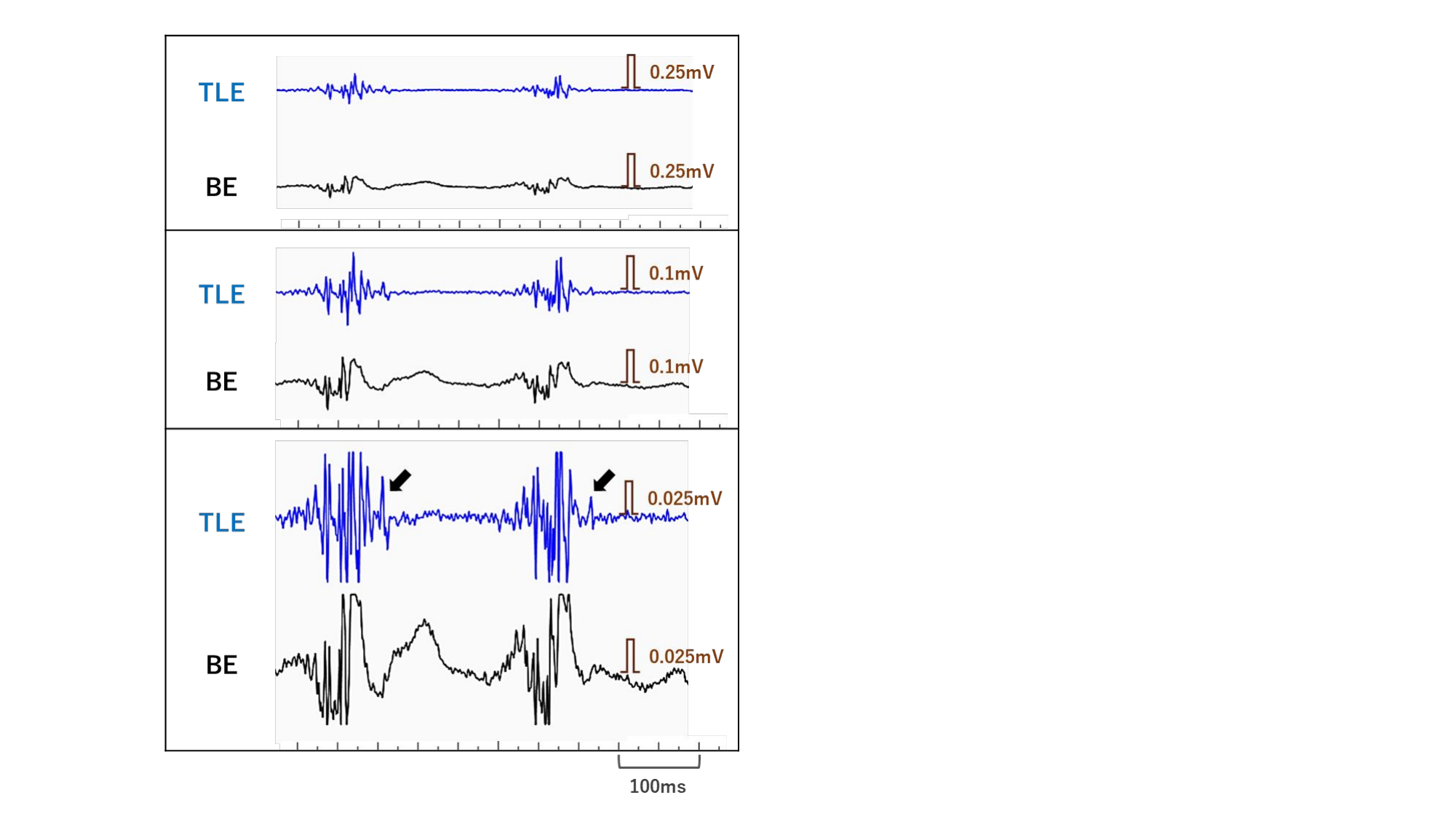

Supplement: Supplementary file 1 — Figure S1. [file JOA3-41-e70101-s002.pptx]
