# Supplementary material for: The signal quality of tripolar Laplacian electrogram compared to bipolar electrogram in cardiac electrophysiology
Source: J Arrhythm. 2025 May 29;41(3):e70101. doi: 10.1002/joa3.70101 (PMC12120260; doi:10.1002/joa3.70101)
Supplement: Supplementary file 2 — Table S1. [file JOA3-41-e70101-s003.docx]

Supplemental table. Mapping data

| **Characteristic** | n=32 |
| --- | --- |
| **Mapping points** | 12,433 ± 1,289 |
| **Low voltage area** | 15 (47%) |
| **Rhythm in mapping** |  |
| **atrial pacing** | 24 (75%) |
| **peri-tricuspid reentrant tachycardia** | 3 (9%) |
| **peri-mitral reentrant tachycardia** | 3 (9%) |
| **focal tachycardia** | 2 (6%) |
