# Supplementary material for: The signal quality of tripolar Laplacian electrogram compared to bipolar electrogram in cardiac electrophysiology
Source: J Arrhythm. 2025 May 29;41(3):e70101. doi: 10.1002/joa3.70101 (PMC12120260; doi:10.1002/joa3.70101)
Supplement: Supplementary file 3 — Data S1. [file JOA3-41-e70101-s001.docx]

**Supplemental figure**

Representative case of atrial tachycardia showing low-amplitude fractionated EGMs at the SC/Ve area. The blue tracing indicates TLE, and the black tracing indicates BE. Raising the resolution from 0.25 mV to 0.025 mV, drift noise became more pronounced on BE, making it difficult to identify the whole EGMs. On TLE, small and fractionated potentials were still visible with a reproducibility (black arrow).

Abbreviations: BE, bipolar electrogram; EGM, electrogram; SC, slow conduction; TLE, tripolar Laplacian electrogram; Ve, vertical.
